# Fruit Size and Structure of Zoochorous Trees: Identifying Drivers for the Foraging Preferences of Fruit-Eating Birds in a Mexican Successional Dry Forest

**DOI:** 10.3390/ani11123343

**Published:** 2021-11-23

**Authors:** R. Carlos Almazán-Núñez, Edson A. Alvarez-Alvarez, Pablo Sierra-Morales, Rosalba Rodríguez-Godínez

**Affiliations:** 1 Laboratorio Integral de Fauna Silvestre (Área de Ornitología), Facultad de Ciencias Químico Biológicas, Universidad Autónoma de Guerrero, Chilpancingo 39090, Guerrero, Mexico; bio_ed19@hotmail.com (E.A.A.-A.); sierra02pix@hotmail.com (P.S.-M.); goretti.merced3@gmail.com (R.R.-G.); 2 Posgrado en Recursos Naturales y Ecología, Facultad de Ecología Marina, Universidad Autónoma de Guerrero, Acapulco 39390, Guerrero, Mexico

**Keywords:** vegetation structure, *Bursera* species, frugivorous birds, legitimate seed dispersers, successional gradient, Balsas river basin

## Abstract

**Simple Summary:**

Tropical dry forests are highly threatened by human activities such as agriculture, livestock, and selective logging. These activities have resulted in fragments of tropical dry forest under different successional stages that negatively affect the interaction between plants and fruit-eating birds. We analyzed the consumption of the fruits of zoochorous trees by birds during the dry season in a tropical dry forest and evaluated whether the horizontal and vertical structure of these trees explains fruit consumption. We also related the bird body mass and fruit size removed from zoochorous trees. We found that the tree structure can influence the visitation of fruit-eating birds, and therefore, the number of fruits consumed as the succession progresses. There was a relationship between fruit sizes and bird body mass in the successional gradient. Our results indicate that structural and fruit traits of zoochorous trees drive foraging preferences of fruit-eating birds, whose interactions in different successional stages can help to reverse the negative effects of fragmentation in tropical dry forests of the study area.

**Abstract:**

Tropical dry forests (TDFs) are affected by land-use changes. These modifications impact their composition and arboreal structure, as well as the availability of food for several bird groups. In this study, we evaluated the foraging preferences in zoochorous trees of fruit-eating birds during the dry season of the year in three successional stages (early, intermediate, and mature) of TDFs in southern Mexico. The fruits of these trees are important in the diet of several birds during the dry season, a period during which food resources are significantly reduced in TDFs. We estimated foliar cover (FC) and foliage height diversity (FHD) of zoochorous trees in 123 circular plots. These variables were recognized as proxies of food availability and tree productivity. Foraging preferences were evaluated at the community level, by frugivore type, and by bird species. We evaluated the effect of the structural variables and the fruit size of zoochorous plants on fruit removal by birds and related the bird body mass and fruit size removed in the successional gradient. A total of 14 zoochorous tree species and 23 fruit-eating bird species were recorded along the successional gradient. Intermediate and mature stages showed greater fruit removal. The birds removed mainly *B. longipes* fruits across the three successional stages. The FHD and fruit size were important drivers in the selection of zoochorous trees and fruit removal by fruit-eating birds. Fruit size and bird body mass were positively related along the successional gradient. The results suggest that fruit removal by fruit-eating birds in the successional gradient can promote the demographic dynamics of several zoochorous tree species, especially of *Bursera* spp. along the TDFs.

## 1. Introduction

Tropical dry forests (TDFs) maintain constant dynamics of land-cover change that are the result of intense anthropogenic activity for agricultural purposes, thus giving rise to mosaics of TDF under different successional stages [1,2]. Anthropogenic changes have disturbed 73% and 66% of primary TDF cover in Mexico and the Americas, respectively [3,4]. These modifications generally influence the composition and foraging preferences of birds [5,6,7,8]. Consequently, anthropogenic disturbances lead to changes in biotic interactions, such as seed dispersal, that are key to maintaining the structure and dynamics of plant populations [9,10]. In TDFs of Mexico, several studies have shown that the population dynamics of woody plant species depend on seed dispersal by frugivorous birds [11,12]. For example, the fruits of a few *Bursera* and *Neobuxbaumia* species are highly removed and dispersed by several bird groups, such as Cardinalidae, Columbidae, Tyrannidae, and Vireonidae [11,12,13,14,15,16].

Marked climatic seasonality and constant anthropogenic disturbances affect fruit removal by birds in TDFs. Fruit removal is related to the fruiting of zoochorous plant species (i.e., plants with diasporas consumed and dispersed by frugivorous animals) [6], which produce fruit asynchronously throughout the year. In addition, TDFs are distributed in patches under different secondary successional stages so that plant composition and fruit provisioning are spatially heterogeneous. For example, in the TDF of southern Pacific Mexico, the arboreal component in the advanced successional stages is usually dominated by the *Bursera* species, which bears fruit mainly during the dry season [17]. Thus, in the initial successional stages, the availability of zoochorous trees is lower, and consequently, several groups of fruit-eating birds also decrease [7,13].

Particular traits of plants and fruits affect the feeding behaviour of bird species in tropical forests, such as nutritional value [18,19,20], harvest size production [21,22] and fruit size [23]. Of these traits, the fruit size determines the species composition and type of frugivore foraging in the zoochorous trees [24,25]. For example, larger fruit tend to be dispersed by large-bodied birds, whereas plant species with small fruits are preferentially selected by small-sized frugivores [26]. Although the negative effect of habitat conversion on large-sized birds is known [27], the relationship between the body size of fruit-eating birds and fruit size of plants along a successional gradient of TDF has not been studied. However, recent studies in different ecosystems have found that the large-bodied frugivorous birds are most affected by forest conversion [27,28]. This bird group has been identified as effective seed dispersers because they remove high volumes of seeds and disperse them over long distances [29]. Therefore, anthropogenic impacts may have significant consequences for ecological dynamics in plant populations [28,29].

In addition to the fruit traits, some structural variables of zoochorous trees, such as foliar cover (FC) and foliage height diversity (FHD), largely determine the abundance and distribution of different bird groups, as these two variables assume higher productivity and a direct relationship with the quantity of propagules removed by birds [30,31,32,33,34,35,36,37]. Overall, FC and FHD are correlated with tree size, and generally, larger-sized trees are found in advanced successional stages with higher values of both structural variables, which can be used as proxies for food availability [38]. Although these variables have been widely recognized as fair predictors to explain bird diversity [30,39,40], they have also rarely been considered in frugivory studies as possible drivers of foraging preferences by birds [41], specifically across the Neotropical dry forests.

In general, a high presence of fruit-eating birds has been observed in the Neotropical TDF in the rainy season [42,43]. Conversely, a higher presence of fruit-eating birds has been observed in the dry forests of the Balsas basin biotic province in southern Mexico during the dry season [7]. The Balsas river basin is co-dominated by *Bursera* spp., which bears fruit during the dry season and is home to many visiting birds [6,7]. In this scenario, knowledge about how birds use distinct structural variables of zoochorous trees, specifically their preference for certain fruits according to their size, has implications on the management, restoration, and conservation of secondary TDFs of the Neotropics. Information on the relationship between birds and native plant species, such as Burseraceae [14] and Cactaceae [44,45], can enhance our understanding of the successional dynamics of TDFs [46].

The main aim of this study is to evaluate the foraging preferences of fruit-eating birds in zoochorous trees in three successional stages of TDFs (early, intermediate, and mature) during the dry season. The study focused on the following questions: (1) What is the successional stage with the highest level of fruit removal by birds? (2) How does the fruit size and structure of zoochorous trees influence the foraging preferences of fruit-eating birds in the successional gradient? (3) Is there a relationship between the birds’ body mass and the size of fruit removed along the successional gradient? To answer these questions, we hypothesize that: (1) Due to greater dominance of zoochorous trees in mature successional stages compared to the early and intermediate stages [47], we expect to find more fruits removed by fruit-eating birds in sites of advanced succession. At these advanced succession sites, we also expect an increase in large-bodied fruit-eating birds, which would not occur in the early seral stages, as this bird group tends to be most affected by habitat conversion. (2) Resource consumption is dependent on food availability. Thus, we expect that fruit-eating bird species will remove more fruits in zoochorous trees with high FC and FHD values in the three successional stages. According to this prediction, we also expect that FHD will have the greatest effect in explaining fruit removal, as this variable is considered a good predictor of bird diversity [30]. (3) Finally, as the selection of zoochorous trees by fruit-eating birds is also dependent on fruit size [29], we expect that the birds’ body mass will positively correlate with the size of fruit removed along the successional gradient.

## 2. Materials and Methods

### 2.1. Description of the Study Area and Sampling Sites

**Figure 1:**
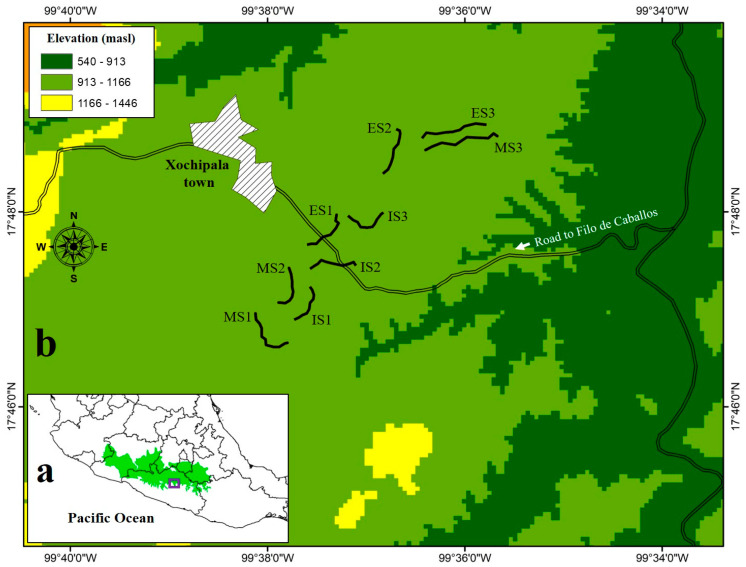
Geographical location of the study area in the Balsas basin, southern Mexico (**a**) and the sampling transects (**b**). The numbers (1–3) represent the three sites of each successional stage: early stages (ES), intermediate stages (IS), and mature stages (MS).

The study area is located in the Balsas basin biotic province in the state of Guerrero, southern Mexico (18° 03′ 46.65″ and 17° 42′ 11.14″ N and 99° 36′ 36.50″ and 99° 35′ 30.46″ W; Figure 1). The orography of the region is rugged, with slopes ranging from 15° to 45°. The average altitude is 1100 m asl. The area has a well-marked climatic regime. The rainy season lasts from June to October, and the dry season from November to May. The predominant climate is semi-warm and subhumid with an annual mean temperature of 23.9 °C and an annual rainfall of 684 mm [48,49]. The main vegetation type is a tropical dry forest in different successional stages, combined with agricultural areas and cattle pastures [47].

The sampling sites represent three successional stages of TDF with distinct vegetation structure and composition [47]:

(1) Early stages (~15 years, ES) are composed of vegetation that arose spontaneously in areas used for livestock and temporal agriculture. Currently, these sites continue to be subjected to species-selective logging; hence, there are scattered trees in rocky soils unsuitable for large-scale agriculture [47]. Some representative species from these sites are *Gliricidia sepium*, *Acacia cochliacantha*, *Ipomoea pauciflora*, *Ceiba aesculifolia*, and *Bursera longipes*.
(2) Intermediate stages (~30–35 years, IS) represent a transition zone between the early and mature forests. These sites were also used for livestock and temporal agriculture for corn and bean production. Nonetheless, to a large extent, they have developed structural and floristic elements from the original vegetation. The dominant species at this stage are *Lysiloma tergemina*, *Exostema caribaeum*, *Bursera longipes*, *B. morelensis*, and *Pterocarpus acapulcensis*. The orography of these sites is not very rugged, with slopes of 15° to 30°.
(3) Mature stages (>50 years, MS) are sites characterized by the presence of typical plant species of mature forests, such as *Bursera* spp., *Desmanthus balsensis*, *Mimosa polyantha*, *M. goldmanii*, and *Pseudosmodingium perniciosum*. These sites were subjected to a clear-cut-and-burn system for agricultural and/or ranching activities, but their abandonment permitted the regeneration of the vegetation. The orography of these sites is rugged, with slopes >30° [47].

Three sites were selected for each successional stage. In each of these successional stages, two sites were selected with 15 plots and one with 11 (41 plots per successional stage and 123 plots in total). The plot numbers among sites of each successional stage varied due to the orographic conditions and accessibility that they presented. Each plot had a 30 m radius (0.28 ha), representing an area of 34.77 ha for the study area.

### 2.2. Fruiting Phenology

The zoochorous tree species in the study area bear fruits mainly during the dry season (Table S1), most of them belonging to the *Bursera* genus. *Pachycereus weberi*, *Capparis* spp., and *Sideroxylon capiri* were other zoochorous species also found to bear fruits. The fruiting period of *P. weberi* and *Capparis* spp. included one month of the dry season, whereas *S. capiri* bore fruits for three months in the same season (Table S1).

### 2.3. Foliar Cover, Foliage Height Diversity, and Fruit Size of Zoochorous Trees

Two perpendicular lines oriented to the four cardinal points were marked with a rope within each plot in the three successional stages to measure the structure of the zoochorous tree species as a proxy for food availability and tree productivity. Zoochorous plants with a diameter-at-breast height (DBH) ≥ 10 cm whose branches intersected with the lines of each plot were identified and measured. Thus, the FC and FHD measures were obtained individually by each tree and expressed on average. Tree foliar cover, or FC (i.e., a horizontal measure defined as the proportion of the soil occupied by the vertical projection of foliage) [50], was estimated with the ellipse formula, using the maximum and minimum length obtained in each tree [50]. Foliar stratification, or FHD, was measured with an optical square marked by two perpendicular axes [51]. Three mirrors were positioned in the square in such a way that a person looking horizontally through the device was able to determine the height of objects above [47]. This procedure was repeated every 1 m along the two perpendicular lines running from the central point toward the four cardinal points. The recorded heights were grouped at 1 m intervals. Finally, the foliage height diversity (i.e., a vertical measure indicating the diversity of foliage heights) [30] was calculated with the Shannon-Wiener index for each zoochorous tree species. As most of the zoochorous trees in the study area shed their foliage during the dry season, the FC and FHD were measured when these trees still had the foliage in order to avoid underestimating the measurements of both structural variables. At the same time, we randomly selected ten zoochorous trees with ripe fruits along the successional gradient. The equatorial diameter of each fruit was measured as a proxy of its size. Although the fruits of *P. weberi* were measured, they were not considered in the subsequent analysis because the fruits of this cactus are usually berries with abundant mesocarp and many seeds. They differ notably from the fruits of the rest of the zoochorous trees, which contain one seed or a maximum of three (i.e., *Capparis* spp).

### 2.4. Foraging Observations in Zoochorous Trees

Foraging observations were performed for eight months (February to May 2011, November and December 2011, and January–February 2012) corresponding to the dry season in the study area. We recorded only those bird species that visited the trees to remove the fruits. The fruit-eating bird species were recorded at each site using a fixed-radius-point count method with a 30-m radius (*n* = 123 point-counts) [52]. Each point was spaced 200 m apart to avoid data duplication. The observations were carried out in the hours of highest bird activity, both in the mornings (07:00 to 11:00 h) and afternoons (16:00 to 19:00 h). Each successional stage was visited 24 times (72 times in total), 12 in the mornings and 12 in the afternoons. The observation time of the birds at each point was 10 min [53]. The order of observation varied between sites to avoid skewing the observations to a single schedule. Different coloured ribbons were placed on the trunk of some species of zoochorous trees (e.g., Burseraceae), representing the increased difficulty of in situ identification, especially when trees shed their foliage during the dry season.

During the surveys, the fruit-eating bird species, the number of individuals, the plants in which they foraged, and the fruit manipulation type of birds were recorded. Fruit-eating birds were grouped into three categories based on direct observations in the field and specialized literature [6,54,55]: potential legitimate dispersers (species that swallow the whole fruit and defecate the seeds away from the parent plant without apparent damage), seed predators (damages the seeds or swallows the whole fruit) and pulp consumers (small birds that eat only the pulp and discard the seed). An observation of foraging was considered positive when the individuals consumed and/or bit the fruits. It was considered a new foraging record when individuals moved towards another branch of the same tree or towards another zoochorous tree to consume fruits.

With the foraging observations, a tree preference index (TPI) [56] was calculated, based on the formula TPI = (O − E)/E, where O = number of times a bird species consumed fruits on a zoochorous tree and E = number of expected foraging observations that would have been observed had the birds selected trees according to their FC or FHD values (i.e., randomly). The parameter E was estimated by multiplying the total number of foraging observations for a given bird species by the FC and FHD of a given tree species. This analysis was performed independently, both with tree foliar cover and foliar height diversity. TPI values close to zero indicate a random use of a tree species, while values significantly higher than zero indicate a preference, and values below zero indicate avoidance [56]. FC and FHD were used as proxies of food availability (i.e., fruits), as both structural variables have been recognized as fair predictors of foraging activity in several groups of terrestrial vertebrates, including fruit-eating birds in tropical forests [57,58]. We also reviewed mean bird body mass recorded in the study area according to the EltonTraits 1.0 database [59].

### 2.5. Data Analysis

The number of fruits removed in the zoochorous tree species was compared between successional stages through a one-way analysis of variance (ANOVA) with Tukey’s HSD *a posteriori* test. Before this analysis, data were transformed to log (x + 1) to fulfil the assumptions of normality and homoscedasticity. We also compared the fruit-eating bird body mass (log10) among the three successional stages using a one-way ANOVA. On the other hand, deviations in foraging preferences by fruit-eating birds from expected occurrence per particular zoochorous tree species were assessed using a chi-square (χ²) goodness-of-fit test of the three successional stages. We evaluated whether the birds foraged on each tree species in proportion to their availability in the study area. For this, we compared observed frequencies of foraging observation of individual species with expected frequencies. This analysis was performed for the fruit-eating-bird community, by fruit-eating-bird type, and by bird species.

To evaluate the effect of the structural variables (i.e., foliar cover and foliage height diversity) and the fruit size of zoochorous plants on fruit removal by birds (i.e., response variable) in the successional gradient, we carried out a generalized linear mixed model (GLMM) with Poisson-type error. Before this analysis, we checked the multicollinearity among the variables using a correlation matrix. The FC and FHD showed a certain correlation (*r* = 0.65), so we only retained FHD, as this variable has been recognized as a good predictor of bird diversity [30]. FHD and fruit size were included as fixed factors, whereas the three successional stages were included as random effects. This procedure was carried out both for all fruit-eating birds and by type of fruit-eating bird. The best model was selected according to the lowest Akaike’s information criterion value (ΔAICc value < 2 between models). We also performed a GLMM to evaluate the relationship between the fruit size and bird body mass along the three successional stages with Gamma-type error distribution and log-link function, including the successional stages as random factor. All statistical analyses were considered significant when *p* ≤ 0.05. These analyses were performed using the *lme4* library [60] in R 4.1.0 [61].

## 3. Results

### 3.1. Foliar Cover, Foliage Height Diversity, and Fruit Size of Zoochorous Trees

*Bursera longipes* and *B. morelensis* had the highest FC values in the three successional stages. *B. longipes* presented the highest FHD values in the early and mature stages. *B. vejarvazquezii* and *B. morelensis* presented the highest FHD values in the intermediate stage. *Pachycereus weberi*, *Sideroxylon capiri*, *Capparis* spp., and *B. longipes* had the largest fruit sizes compared to the rest of the zoochorous trees (Table 1).

### 3.2. Fruit Removal in Zoochorous Trees by Successional Stage

**Figure 2:**
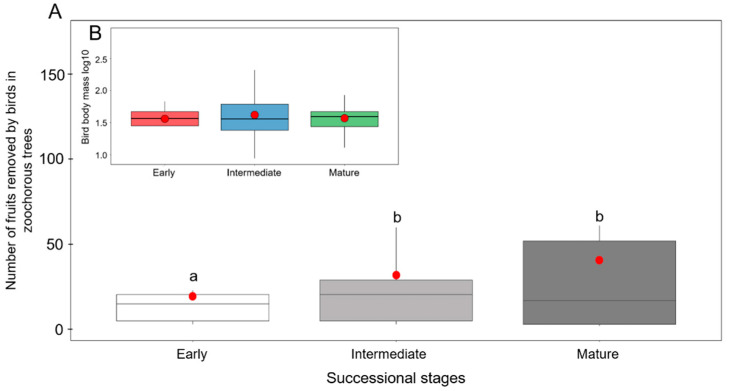
(**A**) Fruit removal in zoochorous trees and (**B**) fruit-eating bird body mass along a successional gradient of TDF in southern Mexico. The red point in each boxplot represents the average value of the removed fruits and bird body mass. Different letters denote significant differences (Tukey; *p* ≤ 0.05).

A total of 23 species of fruit-eating birds foraged in 14 species of zoochorous trees along the successional gradient (Table S2). A greater number of fruits removed was observed in the intermediate and mature stages, compared to the early stage (F_2,22_ = 3.671, *p* = 0.044; Figure 2a). Bird body mass did not vary along the successional gradient (*p* > 0.05; Figure 2b), although the bird composition was not the same in the three stages (Table S2).

### 3.3. Foraging Preferences in Zoochorous Trees by Successional Stage

**Figure 3:**
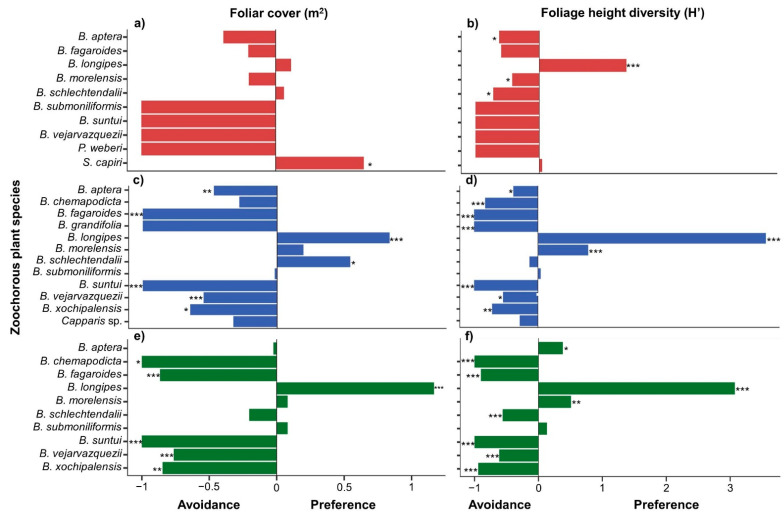
Foraging preferences of fruit-eating birds in a TDF successional gradient in southern Mexico: (**a**,**b**) early stage, (**c**,**d**) intermediate stage, and (**e**,**f**) mature stage. Significant preferences or avoidances are shown with * *p* ≤ 0.05, ** *p* ≤ 0.01, *** *p* ≤ 0.001.

Fruit-eating birds showed a higher foraging preference for *Bursera longipes* than expected by chance in the three successional stages, using both FC and FHD (Figure 3a–f). The TPI obtained using FC showed that fruit-eating birds usually forage on *Sideroxylon capiri* in the early stage and *B. schlechtendalii* in the intermediate stage (Figure 3a,c). However, with FHD, *B. morelensis* was significantly preferred for foraging in the intermediate and mature stages, along with *B. aptera* in this last successional stage (Figure 3d,f).

According to FC, the TPI values by fruit-eating bird type showed that pulp consumers, potential legitimate dispersers, and seed predators fed mainly on *B. longipes* fruit in the intermediate and mature stages (Table S3). In the early stage, potential legitimate dispersers foraged more than expected by chance on *S. capiri*. In the intermediate and mature stages, pulp consumers and seed predators also foraged significantly on *B. schlechtendalii* and *B. vejarvazquezii*, respectively (Table S3). According to the FHD, the TPI values depicted that *B. longipes* and *B. morelensis* were the preferred species for foraging by pulp consumers and potential legitimate dispersers in the three successional stages (Table S4).

At a specific level, most bird species removed fruits from *B. longipes* (Tables S3 and S4). However, a few legitimate dispersers, such as *Melanerpes chrysogenys*, *Calocitta formosa*, and *Icterus pustulatus*, foraged more than expected by chance on *S. capiri*. Pulp consumers, such as *Passerina versicolor*, *P. lechlancherii*, and *Haemorhous mexicanus*, removed small fruits of *B. schlechtendalii* and *B. morelensis*. Conversely, seed predators, such as *Eupsittula canicularis* and *Passerina caerulea*, preferred trees with large fruits, such as *B. longipes*, *B. morelensis*, and *B. vejarvazquezii* (Tables S3 and S4).

### 3.4. Effects of Fruit Size and Structural Variables of Zoochorous Trees on Fruit Removal

The FHD strongly influenced fruit removal both by all fruit-eating birds and by the type of fruit-eating bird along the successional gradient (Table 2). Fruit size also positively affected the fruit removal by all groups of fruit-eating birds, particularly large-bodied birds, such as seed predators and legitimate dispersers (Table 2). It is necessary to highlight that of the total fruit-eating bird species of the study area, only three were seed predators, and four were pulp consumers, which could represent a possible limitation of this analysis.

### 3.5. Relationship between Bird Body Mass and Fruit Size of Zoochorous Trees

**Figure 4:**
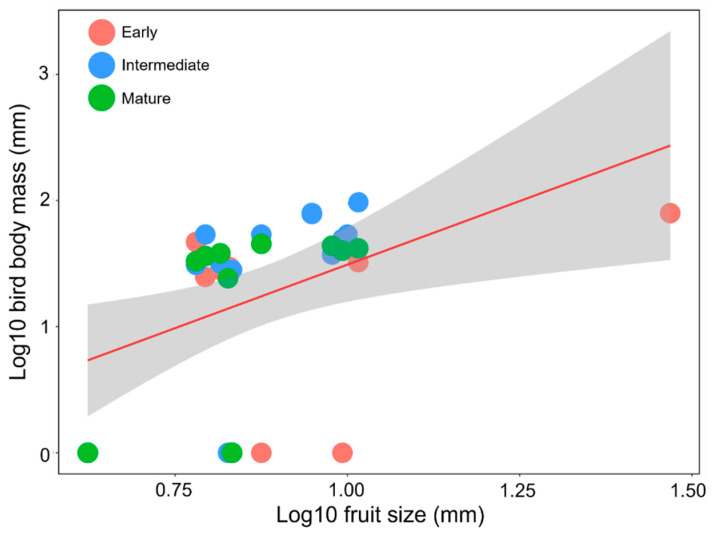
Relationship between bird body mass and fruit size of zoochorous trees along the successional TDF gradient in southern Mexico. The solid line represents a linear regression, and grey colour shows the 95% confidence interval.

For feeding foraging, bird body mass was related to the fruit size of the zoochorous trees along the successional gradient (GLMM: β = 0.079; *p* = 0.031; AIC = 194.4; Figure 4). *Sideroxylon capiri* had the largest fruit size (29.41 mm), and the birds foraging for food on this tree were also large.

## 4. Discussion

Our results show that the greatest number of fruits was removed in the intermediate and mature stages compared to the early stage, confirming the first hypothesis regarding an increase in the fruit removal in advanced successional stages. This pattern has been observed in other studies that have documented that fruit consumption by distinct fruit-eating bird groups is favoured by more structurally complex environments [23,62]. Additionally, several studies conducted in the Mesoamerican tropical regions have suggested that disturbance and/or early seral stages generally decrease the abundance and richness of frugivorous birds [6,34,63], which, in turn, diminishes fruit removal [64,65] and seed dispersal [10,13,66,67]. Nevertheless, it has also been shown that in highly disturbed sites, the presence of some zoochorous trees plays a relevant role in attracting some groups of fruit-eating birds [45,46,68,69] that contribute to seed dispersal and, in turn, help in the restoration of secondary dry tropical ecosystems [46,69,70].

It is important to highlight that there were no differences in bird body mass among the successional stages, which means that several fruit-eating birds of different sizes are indistinctly foraging in the zoochorous trees along the successional gradient. However, bird composition could explain fruit removal along the successional gradient. For example, some large- and medium-bodied bird species, such as *Ortalis poliocephala*, *Eupsittula canicularis*, *Pheucticus melanocephalus*, and *Momotus mexicanus*, were observed foraging only in the intermediate and mature stages. These fruit-eating birds require more structurally complex forests to feed and carry out other functions, such as reproduction, refuge, and rest [46,71,72,73], which cannot be carried out in the early seral stages. This confirms that forest disturbance tends to affect certain bird groups, particularly those that are larger and require forest conditions to survive [29]. These birds have been documented to have a higher risk of extinction [18], leading to further changes in key ecological processes (i.e., seed dispersal) for maintaining the dynamics of the plant communities [74]. Other bird species of larger size (*Calocitta formosa*, *Melanerpes chrysogenys*) also have more general habitat requirements, promoting seed dispersal in different successional stages [6]. This underlies that all fruit-eating birds, particularly the large and medium birds, should be protected from the impact of human activities [75].

In general, fruit-eating birds mainly removed the fruits of *Bursera* spp. with high FC and FHD values, with *B. longipes* and *B. morelensis* presenting the most complex vertical and horizontal structures in the three successional stages. In addition, the FHD strongly and positively influenced fruit removal of both zoochorous arboreal species, confirming our second hypothesis that FHD would be the most significant variable for fruit removal along the successional gradient. This result could be due to structural variables related to food availability for the distinct fruit-eating bird groups [6,33,40]. This means that birds were found feeding on fruits mainly from more productive trees. Several bird species, such as *Eupsittula canicularis*, *Melanerpes chrysogenys*, *Pheucticus melanocephalus*, *Myiarchus tuberculifer*, and *M. tyrannulus*, foraged in zoochorous trees with higher FC and FHD. *Bursera* fruits tend to be highly removed by different bird groups [12,13,14], which could explain the positive relationship between the vertical structure of zoochorous trees and fruit-eating bird species across the successional gradient.

Fruit size also positively affected fruit removal by all groups of fruit-eating birds, particularly seed predators and potential legitimate dispersers; the latter mainly consumed fruits of *B. longipes* and *B. morelensis* in all successional stages. These two tree species are abundant in the study area and seem to adapt to varying environmental conditions of the successional gradient, including disturbed areas [17,47]. In fact, fruit size has been identified as a trait that drives fruit removal and seed dispersal [76,77,78], particularly of several *Bursera* species [12,13,79]. Thus, the interaction between the fruit size of these trees and potential legitimate dispersers increases the probability of germination and the establishment of seedlings in the TDF of the study area. The effectiveness of the legitimate dispersers in the seed dispersal, particularly those of the Tyrannidae family, has been shown in several *Bursera* species, such as *B. morelensis* [80] and *B. longipes* [12].

*Sideroxylon capiri* was another tree widely preferred by potential legitimate dispersers in the early seral stages. Its large fruits (29.41 mm) and the large-bodied fruit-eating birds that foraged on this tree species highlight the importance of fruit size in the removal, and consequently, seed dispersal of the zoochorous plants. This tree species presents an important vertical stratification, reaching up to 25 m in isolated, disturbed secondary forests [81,82], which explains its high FHD values in the early stage. In the study area, this high vertical complexity contrasts with the foliar or horizontal cover, which presented lower values in relation to the FHD, so the foraging preference values were significant with FC. In addition, the high foraging preference on *S. capiri* is related to its being sub-deciduous (unlike most trees and shrubs in the TDF), maintaining its foliage during the dry-season fruiting period [82]. In early succession stages, the foliage and fruits of these sub-deciduous trees permit fruit-eating birds to feed for a long time during the dry season, causing a reduction in the species’ energy, which, in turn, would reduce their movement to other sites in search of food [83]. In addition, the foliage of these trees protects fruit-eating birds from predators during the dry season. In this season, birds are usually more visible due to the defoliation of most plants, and therefore, more exposed to predation [20]. This is especially true in the early successional stages [70], in which the density of the trees and shrubs decreases significantly [47]. Therefore, the probability of germination and the establishment of seedlings are the two most critical phases in the development of plants in the TDF [84]. This seed-dispersal service from legitimate disperser birds could promote the restoration of secondary dry forests throughout the Neotropics [12,47].

As we hypothesized, the fruit size of zoochorous plants was positively related to the body mass of the fruit-eating birds along the successional gradient. For example, small-bodied pulp consumers, such as *Haemorhous mexicanus*, *Passerina versicolor*, and *P. leclancherii*, removed small-sized fruits in *B. aptera* and *B. schlechtendalii* [78], while large-bodied legitimate dispersers such as *Calocitta formosa*, *Melanerpes chrysogenys*, *Ortalis poliocephala*, and *T. verticalis* consumed large-sized fruits in *Sideroxylon capiri*, *B. longipes*, and *Capparis* spp. This shows that the selection of zoochorous trees by fruit-eating birds is highly dependent on plant fruit size, as previously described [77,85]. Although this is not surprising, it has important ecological implications because the effectiveness of seed dispersal by fruit-eating birds depends, to a great extent, on their body mass [74,76], which highlights the importance of morphological traits in the mutualistic frugivory networks in tropical environments [85]. In addition, our results show that the TDF successional gradient does not appear to affect the relationship between fruit size and body mass in fruit-eating birds. Despite the change in species composition along the successional gradient and the absence of some large frugivores (i.e., *Ortalis poliocephala*) in early seral stages, other large generalist birds, such as *Calocitta formosa*, were common at these sites. These generalist birds tend to be functionally redundant in seed-dispersal services [86,87], which suggests that at each seral stage of the study area, this ecological service may be being provided. However, the effectiveness of seed dispersal by these species is unknown, so studies focused on evaluating this aspect will be required in the future.

## 5. Conclusions

The results show that FHD is an important structural variable related to food availability and productivity that seems to influence the foraging of distinct fruit-eating bird groups. Fruit size is an important driver in the selection and fruit removal of zoochorous tree species by fruit-eating birds. This highlights that seed dispersal by fruit-eating birds is related to morphological traits (i.e., body mass) in the mutualistic frugivory networks in Neotropical secondary TDFs. However, although TDFs are dominated by plants with dispersal syndromes such as anemochorous and autochorous [2], in this study, we showed that the relationship between these morphological and structural traits of zoochorous trees and fruit-eating birds in this ecosystem is very close [12,79]. Along the successional gradient of the study area, this relationship is closer in the dry season, a period in which most *Bursera* spp. bear fruit [7,88]. In particular, a significant interaction was observed between different fruit-eating bird groups with *Bursera* species. The presence of these bird groups in different successional stages can contribute to the passive restoration of TDF in the study area and the demographic dynamics of *Bursera* spp., an arboreal species that must be conserved due to its high levels of endemism in the Balsas river basin and southern Mexico [17,89]. This is important because 63% of primary TDFs have been replaced by secondary vegetation in the Balsas basin [90]. Given the degree of anthropogenic threat of TDFs in the Neotropics [48], such studies could help to redirect management, conservation, and restoration efforts of this highly threatened ecosystem at national and global levels.

## Figures and Tables

**Table 1:** Values of foliar cover, foliage height diversity, and fruit size of zoochorous trees along the successional gradient of TDF in southern Mexico. The cells with a hyphen mean that the tree species were not present in successional stages.

Plant Species	Fruit Size (mm)	Foliar Cover (m^2^)			Foliage Height Diversity (H’)		
		Early	Intermediate	Mature	Early	Intermediate	Mature
*Bursera aptera*	6.5	73.27	1241.53	1114.4	1.04	1.84	1.84
*Bursera chemapodicta*	7.5	-	144.25	123.11	-	1.04	1.58
*Bursera fagaroides*	6.7	37.47	409.67	463.04	0.64	1.84	1.47
*Bursera grandifolia*	8.9	-	22.46	-	-	1.57	-
*Bursera longipes*	10.4	468.51	2763.64	1669.78	1.9	1.91	2.08
*Bursera morelensis*	6.2	139.85	1736.57	1179.67	1.68	2	1.98
*Bursera schlechtendalii*	6.0	21.07	581.74	446.22	0.69	1.79	1.89
*Bursera submoniliformis*	7.5	6.83	1055	819.84	0.1	1.71	1.81
*Bursera suntui*	4.2	17.17	460.39	154.23	0.1	1.77	1.55
*Bursera vejarvazquezii*	9.8	8.71	1848.18	1410.25	0.1	2.02	2.02
*Bursera xochipalensis*	9.5	-	489.52	272.22	-	1.06	1.68
*Capparis* spp.	10	-	61.51	-	-	0.1	-
*Pachycereus weberi*	41.9	11.32	-	-	0.1	-	-
*Sideroxylon capiri*	29.4	81.07	-	-	1.11	-	-

**Table 2:** Effects of fruit size and structural variables (FC and FHD) on fruit removal from 14 zoochorous tree species according to the best GLMM in a TDF successional gradient in southern Mexico. Standard error (SE), Akaike’s information criterion (AIC) and significance *p*-value (** *p* ≤ 0.01, *** *p* ≤ 0.001) are shown.

Variables	Estimate	SE	*Z*-Value	*p*-Value	AIC
Fixed effects	All fruit-eating birds				
Foliage height diversity	3.83	0.18	20.57	***	672.64
Fruit size	0.12	0.01	12.78	***	
	Potential legitimate dispersers				
Foliage height diversity	2.65	0.17	15.62	***	560.58
Fruit size	0.11	0.01	10.20	***	
	Seed predators				
Foliage height diversity	7.41	0.91	8.12	***	110.94
Fruit size	0.19	0.05	3.97	**	
	Pulp consumers				
Foliage height diversity	4.41	0.61	7.23	***	180.43
Fruit size	0.09	0.03	3.19	**	

## Data Availability

The data that support the findings of this study are available from the corresponding author.

## Supplementary Materials

The following are available online at https://www.mdpi.com/article/10.3390/ani11123343/s1, Table S1: Monthly fruiting phenology of zoochorous tree species in a TDF of southern Mexico. The space between the dotted lines corresponds to the rainy season of the year, while the rest of the space corresponds to the dry season. Months: January (Ja), February (Fe), March (Ma), April (Ap), May (My), June (Jn), July (Ju), August (Au), September (Se), October (Oc), November (No), December (De), Table S2: Composition of fruit-eating birds along the successional gradient of TDF in southern Mexico. Type of frugivore: pulp consumer (PulC), seed predator (SeedP), legitimate disperser (LegD). Successional stage: early stage (ES), intermediate stage (IS), mature stage (MS). The nomenclature and systematic arrangement of bird species follow the guidelines of the American Ornithological Society [91], Table S3: Foraging preferences based on the coverage of zoochorous trees by group of frugivore and by species of bird for each of the successional stages. Significant preferences (+) or avoidances (−) are shown with: ± *p* < 0.05, ± ± *p* < 0.01, ± ± ± *p* < 0.001. Type of frugivore: pulp consumer (PulC), seed predator (SeedP), legitimate disperser (LegD). The complete names of the zoochorous tree species and fruit-eating bird species are shown in Table S1 and S2, respectively, Table S4: Foraging preferences based on the foliage height diversity of zoochorous trees by group of frugivore and by species of bird for each of the successional stages. Significant preferences (+) or avoidances (−) are shown with: ± *p* < 0.05, ± ± *p* < 0.01, ± ± ± *p* < 0.001. Type of frugivore: pulp consumer (PulC), seed predator (SeedP), legitimate disperser (LegD). The complete names of the zoochorous tree species and fruit-eating bird species are shown in Tables S1 and S2, respectively.
