# Supplementary material for: Fruit Size and Structure of Zoochorous Trees: Identifying Drivers for the Foraging Preferences of Fruit-Eating Birds in a Mexican Successional Dry Forest
Source: Animals (Basel). 2021 Nov 23;11(12):3343. doi: 10.3390/ani11123343 (PMC8697955; doi:10.3390/ani11123343)
Supplement: Supplementary file 1 [file animals-11-03343-s001.zip › animals-1406430-supplementary.pdf]

animals

MDPI

Supplementary Materials

Fruit Size and Structure of Zoochorous Trees: Identifying Drivers for the Foraging Preferences of Fruit-Eating Birds in a Mexican Successional Dry Forest

R. Carlos Almazán-Núñez [1,*], Edson A. Alvarez-Alvarez [1,2], Pablo Sierra-Morales [1,2] and Rosalba Rodríguez-Godínez [1,2]

[1] Laboratorio Integral de Fauna Silvestre (Área de Ornitología), Facultad de Ciencias Químico Biológicas, Universidad Autónoma de Guerrero, Chilpancingo 39090, Guerrero, Mexico; bio_ed19@hotmail.com (E.A.A.-A.); sierra02pix@hotmail.com (P.S.-M.); goretti.merced3@gmail.com (R.R.-G.)

[2] Posgrado en Recursos Naturales y Ecología, Facultad de Ecología Marina, Universidad Autónoma de Guerrero, Acapulco 39390, Guerrero, Mexico

* Correspondence: rcarlos.almazan@gmail.com; Tel.: +52-747-105-6697

Citation: Almazán-Núñez, R.C.; Alvarez-Alvarez, E.A.; Sierra-Morales, P.; Rodríguez-Godínez, R. Fruit Size and Structure of Zoochorous Trees: Identifying Drivers for the Foraging Preferences of Fruit-Eating Birds in a Mexican Successional Dry Forest. *Animals* **2021**, *11*, 3343. https://doi.org/10.3390/ani11123343

Academic Editors: Jesús Domínguez and Sandra Goded

Received: 18 September 2021
Accepted: 15 November 2021
Published: 23 November 2021

Publisher's Note: MDPI stays neutral with regard to jurisdictional claims in published maps and institutional affiliations.

Copyright: © 2021 by the authors. Licensee MDPI, Basel, Switzerland. This article is an open access article distributed under the terms and conditions of the Creative Commons Attribution (CC BY) license (http://creativecommons.org/licenses/by/4.0/).

Table S1. Monthly fruiting phenology of zoochorous tree species in a TDF of southern Mexico. The space between the dotted lines corresponds to the rainy season of the year, while the rest of the space corresponds to the dry season. Months: January (Ja), February (Fe), March (Ma), April (Ap), May (My), June (Jn), July (Ju), August (Au), September (Se), October (Oc), November (No), December (De).

Family	Plant species	Species code	Months											
			**Ja**	**Fe**	**Ma**	**Ap**	**Ma**	**Jn**	**Ju**	**Au**	**Se**	**Oc**	**No**	**De**
Burseraceae	*Bursera aptera*	Bap	■	■	■	■	■							■
Burseraceae	*Bursera fagaroides*	Bfa	■	■	■	■	■							
Burseraceae	*Bursera grandifolia*	Bgr	■	■	■	■	■							
Burseraceae	*Bursera longipes*	Blo	■	■	■	■	■						■	■
Burseraceae	*Bursera schlechtendalii*	Bsc	■	■	■	■	■							■
Burseraceae	*Bursera xochipalensis*	Bxo	■	■	■	■	■							
Burseraceae	*Bursera suntui*	Bsn		■	■	■	■							
Burseraceae	*Bursera vejarvazquezii*	Bve										■	■	
Burseraceae	*Busera morelensis*	Bmo	■	■	■	■	■							
Burseraceae	*Busera submoniliformis*	Bsu										■	■	
Burseraceae	*Bursera chemapodicta*	Bch			■	■	■							
Cactaceae	*Pachycereus weberi*	Pwe					■	■						
Capparaceae	*Capparis* spp.	Csp					■	■						
Sapotaceae	*Sideroxylon capiri*	Sca	■	■										■

Table S2. Composition of fruit-eating birds along the successional gradient of TDF in southern Mexico. Type of frugivore: pulp consumer (PulC), seed predator (SeedP), legitimate disperser (LegD). Successional stage: early stage (ES), intermediate stage (IS), mature stage (MS). The nomenclature and systematic arrangement of bird species follow the guidelines of the American Ornithological Society [91].

Family	Bird species	English name	Species code	Type of frugivore	Successional stages		
					ES	IS	MS
Cracidae	*Ortalis poliocephala*	West Mexican Chachalaca	Ortpol	LegD		x	x
Momotidae	*Momotus mexicanus*	Russet-crowned Motmot	Mommex	LegD		x	
Picidae	*Melanerpes chrysogenys*	Golden-cheeked Woodpecker	Melchr	LegD	x	x	
	Melanerpes hypopolius	Gray-breasted Woodpecker	Melhyp	LegD			x
Psittacidae	*Eupsittula canicularis*	Orange-fronted Parakeet	Eupcan	SeedP		x	x
Tyrannidae	*Myiarchus tuberculifer*	Dusky-capped Flycatcher	Myitub	LegD		x	x
	Myiarchus cinerascens	Ash-throated Flycatcher	Myicin	LegD	x	x	x
	Myiarchus nuttingi	Nutting's Flycatcher	Myinut	LegD		x	
	Myiarchus tyrannulus	Brown-crested Flycatcher	Myityr	LegD	x	x	x
	Myiodynastes luteiventris	Sulphur-bellied Flycatcher	Myilut	LegD	x		x
	Tyrannus vociferans	Cassin's Kingbird	Tyrvor	LegD		x	x
	Tyrannus verticalis	Western Kingbird	Tyrver	LegD	x	x	x
	Tyrannus forficatus	Scissor-tailed Flycatcher	Tyrfor	LegD			x
Corvidae	*Calocitta formosa*	White-throated Magpie-Jay	Calfor	LegD	x	x	
Fringillidae	*Haemorhous mexicanus*	House Finch	Haemex	PulC		x	
	Spinus psaltria	Lesser Goldfinch	Spipsa	PulC	x	x	x
Icteridae	*Icterus wagleri*	Black-vented Oriole	Ictwag	LegD		x	x
	Icterus pustulatus	Streak-backed Oriole	Ictpus	LegD	x	x	
Cardinalidae	*Piranga ludoviciana*	Western Tanager	Pirlud	LegD		x	x
	Pheucticus melanocephalus	Black-headed Grosbeak	Phemel	SeedP			x
	Passerina caerulea	Blue Grosbeak	Paecae	SeedP		x	x
	Passerina leclancherii	Orange-breasted Bunting	Paslec	PulC			x
	Passerina versicolor	Varied Bunting	Pasver	PulC	x	x	x

Table S3. Foraging preferences based on the coverage of zoochorous trees by group of frugivore and by species of bird for each of the successional stages. Significant preferences (+) or avoidances (-) are shown with: ± $p < 0.05$, ± ± $p < 0.01$, ± ± ± $p < 0.001$. Type of frugivore: pulp consumer (PulC), seed predator (SeedP), legitimate disperser (LegD). The complete names of the zoochorous tree species and fruit-eating bird species are shown in Table S1 and S2, respectively.

**Bird species**	**Early**					**Intermediate**										**Mature**									
	**Bap**	**Bfa**	**Bmo**	**Bsc**	**Sca**	**Bap**	**Bch**	**Bfa**	**Blo**	**Bmo**	**Bsc**	**Bsu**	**Bsn**	**Bve**	**Csp**	**Bap**	**Bch**	**Bfa**	**Blo**	**Bmo**	**Bsc**	**Bsu**	**Bsn**	**Bve**	**Bxo**
Pasver			+++								+++							++			+++				
Paslec																			+		+++				
Haemex						++			++																
Spipsa									+	++									+++						
PulC									+++		++	-		--		-			+++	-	++			-	
Eupcan									++					++					+++					+	
Pascae									+++											+++	++				
Phemel																									
SeedP									+++	-				+		-			+++			+			
Ortpol											+														
Melchr					+++					++		++			+++										
Melhyp																				+++					
Myicin	+	+					+++		++					-					+++	-				--	
Myitub									+++										++						
Myityr																++									
Myinut									+	+															
Myilut				+++															+++						
Tyrver												+++		---				-	+++	++	-			---	
Tyrvoc									++		+++								+++			-		--	
Tyrfor																						+++			
Calfor					+++				+++																
Pirlud														+++						+++					
Ictwag										+++									+++						
Ictpus					++						+++			-											
Mommex																									
LegD					+	--		-	+++	+			---	---			-	---	+++				-	---	--

Table S4. Foraging preferences based on the foliage height diversity of zoochorous trees by group of frugivore and by species of bird for each of the successional stages. Significant preferences (+) or avoidances (-) are shown with: ± $p < 0.05$, ± ± $p < 0.01$, ± ± ± $p < 0.001$. Type of frugivore: pulp consumer (PulC), seed predator (SeedP), legitimate disperser (LegD). The complete names of the zoochorous tree species and fruit-eating bird species are shown in Table S1 and S2, respectively.

**Bird species**	**Early**			**Intermediate**												**Mature**									
	**Blo**	**Bmo**	**Sca**	**Bap**	**Bch**	**Bfa**	**Bgr**	**Blo**	**Bmo**	**Bsc**	**Bsu**	**Bsn**	**Bxo**	**Bve**	**Csp**	**Bap**	**Bch**	**Bfa**	**Blo**	**Bmo**	**Bsc**	**Bsn**	**Bsu**	**Bve**	**Bxo**
Pasver		+++						++		+++								+			+++				
Paslec																			+++						
Haemex	+++			++				+																	
Spipsa	+++							+	+++										+++						
PulC	+++							+++						-					+++						
Eupcan								+++						+++					+++						
Pascae																				+++					
Phemel																			++					++	
SeedP								+++						+++		-			+++						
Ortpol								+++																	
Melchr			+++						+++		++				+++	++			++						
Melhyp																			+++						
Myicin			+					+++						-		+			+++	-				-	
Myitub								+++											+++						
Myityr	+++							+++	++							+++	-		+++			-			
Myinut								+++	++																
Myilut	++	-																	+++						
Tyrver	+++					-	-	+++		-		-		--			-	-	+++	+++	--	-	+	--	--
Tyrvoc				-				+++		+++							-		+++			-	-	-	-
Tyrfor																							+++		
Calfor			+++					+++																	
Pirlud								+++												+++					
Ictwag	+++								+++										+++						
Ictpus	++							+++								++			+++	+	-				
Mommex								++																	
LegD	+++	+		-	++	---	---	+++	+++			---	-	--		+++	---	---	+++	+++	---	---		---	---
